# hBcl2 overexpression in BMSCs enhances resistance to myelin debris-induced apoptosis and facilitates neuroprotection after spinal cord injury in rats

**DOI:** 10.1038/s41598-024-52167-4

**Published:** 2024-01-21

**Authors:** Dasheng Tian, Xingyu You, Jianan Ye, Gan Chen, Hang Yu, Jianwei Lv, Fangli Shan, Chao Liang, Yihui Bi, Juehua Jing, Meige Zheng

**Affiliations:** 1 Department of Orthopaedics, The Second Affiliated Hospital of Anhui Medical University, Hefei, 230601 China; 2 Institute of Orthopaedics, Research Center for Translational Medicine, The Second Affiliated Hospital of Anhui Medical University, Hefei, 230601 China

**Keywords:** Stem cells, Medical research, Neurology

## Abstract

After spinal cord injury (SCI), the accumulation of myelin debris at the lesion exacerbates cell death and hinders axonal regeneration. Transplanted bone marrow mesenchymal stem cells (BMSCs) have been proven to be beneficial for SCI repair, but they are susceptible to apoptosis. It remains unclear whether this apoptotic process is influenced by myelin debris. Here, we constructed rat BMSCs overexpressing human B-cell lymphoma 2 (hBcl2) alone (hBcl2 group), BMSCs overexpressing hBcl2 with an endoplasmic reticulum-anchored segment (hBcl2-cb) (cb group), and a negative control group (NC group) for transplantation in this study. Immunocytochemistry staining validated the successful expression of hBcl2 in BMSCs within the hBcl2 group and cb group. All BMSCs from each group exhibited the ability to phagocytize myelin debris. Nevertheless, only BMSCs derived from the hBcl2 group exhibited heightened resistance to apoptosis and maintained prolonged viability for up to 5 days when exposed to myelin debris. Notably, overexpression of hBcl2 protein, rather than its endoplasmic reticulum-anchored counterpart, significantly enhanced the resistance of BMSCs against myelin debris-induced apoptosis. This process appeared to be associated with the efficient degradation of myelin debris through the Lamp1^+^ lysosomal pathway in the hBcl2 group. In vivo, the hBcl2 group exhibited significantly higher numbers of surviving cells and fewer apoptotic BMSCs compared to the cb and NC groups following transplantation. Furthermore, the hBcl2 group displayed reduced GFAP^+^ glial scarring and greater preservation of NF200^+^ axons in the lesions of SCI rats. Our results suggest that myelin debris triggers apoptosis in transplanted BMSCs, potentially elucidating the low survival rate of these cells after SCI. Consequently, the survival rate of transplanted BMSCs is improved by hBcl2 overexpression, leading to enhanced preservation of axons within the injured spinal cord.

## Introduction

Spinal cord injury (SCI) is a devastating disease, frequently leading to permanent impairment of motor, sensory, and autonomic functions^1^. Following SCI, there is a persistent incidence of pathological demyelination both at the lesion epicenter and in the surrounding axons, resulting in the accumulation of significant quantities of myelin debris^2^. This accumulation not only hinders axon regrowth but also triggers an inflammatory response, exacerbating the infiltration of inflammatory cells in the damaged microenvironment and impeding tissue repair^2–4^.

Recent studies have indicated that transplantation of bone marrow mesenchymal stem cells (BMSCs) could effectively facilitate functional recovery via direct differentiation and paracrine actions after SCI^5,6^. Chizuka Ide et al. proved that the beneficial effects of BMSC transplantation on tissue repair, axonal outgrowth, and locomotor improvement in SCI rats^7–9^. However, a significant limitation of BMSCs transplantation in SCI treatment is the low survival rate of transplanted BMSCs at the injury site^10,11^. We propose that BMSCs are prone to apoptosis after transplantation, which may be influenced by the presence of myelin debris. Therefore, a crucial challenge is to increase the survival capacity of BMSCs in the damaged microenvironment and reduce BMSCs apoptosis after transplantation.

B-cell lymphoma-2 (Bcl2) plays a crucial role in inhibiting apoptosis by binding to pro-apoptotic proteins, thereby preventing the permeability of the mitochondrial outer membrane and the release of cytochrome C from the mitochondria to the cytoplasm^12,13^. When Bcl2 localizes to the endoplasmic reticulum, it is also involved in the regulation of neural differentiation, and its overexpression has been shown to upregulate the expression of neural differentiation-associated genes^14^. As we previously reported, the long-term survival of BMSCs in nerve transplants is closely associated with the upregulation of Bcl2^15^.

In this study, we successfully constructed two types of BMSCs for transplantation in a rat SCI model: BMSCs overexpressing human Bcl2 (hBcl2) and BMSCs overexpressing hBcl2 with an endoplasmic reticulum-anchored segment (hBcl2-cb). Our results demonstrate that the overexpression of hBcl2, rather than hBcl2-cb, significantly enhances the resistance of BMSCs against myelin debris-induced apoptosis, improves the survival rate of transplanted BMSCs, and promotes axonal preservation in the injured spinal cord of rats.

## Materials and methods

### Animals

All animal experiments were carried out in accordance with institutional animal care protocols and were authorized by Anhui Medical University Ethical Committee (No. LLSC20160052) starting from March 1st, 2018. Adult female Sprague–Dawley (SD) rats (7–8 weeks old and weighing 200–250 g) were supplied from Experimental Animal Center of Anhui Medical University (No. SCXK (Wan) 2017-001). Animals were housed in a room with a 12-h light/dark cycle at 23–25 °C. All animals had free access to water and food. They were randomized to subsequent experiments as random.

### Experimental design

For this study, BMSCs overexpressing hBcl2 (referred to as the hBcl2 group), BMSCs overexpressing hBcl2 with an endoplasmic reticulum-anchored segment (hBcl2-cb) (referred to as the cb group), and a negative control group (referred to as the NC group) were constructed by lentivirus. The expression of hBcl2 and its effect on BMSCs' phagocytosis of myelin debris and post-transplantation survival were verified using immunocytochemistry staining. Furthermore, the relationship between the anti-apoptotic ability of BMSCs overexpressing hBcl2 and the lysosomal pathway was explored. Finally, the effect of BMSCs overexpressing hBcl2 on functional recovery in rats with spinal cord injury was observed.

### Isolation and characterization of BMSCs

The isolation and phenotypic characterization of rat BMSCs were carried out according to previously established protocols^16^. Five adult female SD rats were used for BMSCs isolation. BMSCs suspensions were extracted from the distal femur and proximal tibia of the rats by repeatedly flushing the medullary cavity with Dulbecco's Modified Eagle’s Medium (DMEM) containing 10% fetal bovine serum (FBS) and 1% Penicillin–Streptomycin (Gibco, USA). The total cell suspension was then cultured at 37 °C with 5% CO_2_ in a humidified incubator. The culture media was changed every 3 days to remove the non-adherent cells. Cells from passages 3 to 6 were collected for subsequent experiments.

The expression of CD29 (ab218273-PE, abcam, United Kingdom), CD90 (ab25322-APC, abcam, United Kingdom), CD34 (ab134207-PE, abcam, United Kingdom), and CD45 (ab27287-FITC, abcam, United Kingdom) in BMSCs was detected by flow cytometry (FACSCanto™, USA) for BMSCs phenotype characterization. Chondrogenic, osteogenic, and adipogenic differentiation were performed to determine the stem cell’s ability of BMSCs by using a three-line differentiation kit (Cyagen, China) following the manufacturer's instructions.

The following media were used to confirm the multipotential differentiation ability of rat BMSCs: (1) adipogenic differentiation medium (10% FBS, 1% Glutamine, 0.1% Insulin, 0.1% Dexamethasone, 0.1% Rosiglitazone, 1% Penicillin-Streptomycin, high-glucose DMEM); (2) chondrogenic differentiation medium (0.3% Ascorbate, 1% ITS + Supplement, 0.1% Sodium Pyruvate, 0.1% Proline, 1% TGF-3, 0.01% Dexamethasone, high-glucose DMEM); and (3) osteogenic differentiation medium (10% FBS, 1% Glutamine, 1% Glycerophosphate, 0.2% Ascorbate, 0.01% Dexamethasone, 1% Penicillin–Streptomycin, high-glucose DMEM). The media were refreshed every 3 days. Alizarin Red staining was used to detect osteocytes, Oil Red O staining for adipocytes, and Alcian Blue staining for chondrocytes.

### Construction of hBcl2 overexpressing BMSCs by lentivirus

The sequence of cDNA of hBcl2 (Plasmid #17999), hBcl2-cb (Plasmid #18000) containing an endoplasmic reticulum-anchored segment cytochrome b5, were obtained from Addgene. Lentiviral vectors, including LV5-hBcl2-GFP, LV5-hBcl2-cb-GFP, and the empty lentiviral control LV5-GFP, were synthesized by GenePharma. Lentiviruses were produced by co-transfecting these lentiviral vectors individually with the packaging plasmids (pGag/Pol, pRev, pVSV-G) into 293T cells using Lipofiter (GenePharma, China).

BMSCs were seeded in sterile petri dishes (6 cm) and allowed to reach 50% confluency. Lentivirus-containing medium (multiplicity of infection = 15) combined with polybrene (5 μg/mL; GenePharma, China) was added for lentiviral infection, according to a previously published protocol^16^. Fresh medium was added to the culture medium after 24 h. Stably-infected BMSCs were obtained after 2 weeks of puromycin (2 μg/mL, Sigma, USA) selection. The normal uninfected BMSCs were used as negative control. The BMSCs successfully infected with the lentiviral vector LV5-hBcl2-GFP were designated as the hBcl2 overexpressing BMSCs group (hBcl2 group). BMSCs infected with the LV5-hBcl2-cb-GFP vector, were referred to as the hBcl2-cb group (cb group). BMSCs infected with the empty vector LV5-GFP were labeled as the NC group. Western blot analysis and Immunocytochemistry were applied to detect the expression levels of hBcl2 in each group.

### TUNEL analysis

BMSCs from all groups were washed with PBS and then fixed using 4% paraformaldehyde (Biosharp, China). The terminal transferase-mediated dUTP fluorescein nick-end labeling (TUNEL) assay was performed using the Cell Apoptosis Detection Kit (elabscience, China) in accordance with the manufacturer’s protocol. The percentage of apoptotic cells was calculated by the proportion of TUNEL^+^ cells in total cells under fluorescence microscope (Zeiss, Germany). Three visual fields were randomly selected from each group for analysis.

### Isolation of myelin debris

The animal experiments were authorized by Anhui Medical University Ethical Committee (No. LLSC20160052) starting from March 1st, 2018. Myelin debris was isolated from the brains of healthy C57BL/6 mice aged 8–12 weeks (n = 10) using sucrose density gradient centrifugation at 100,000 *g*, as described previously^17^. The extracted myelin mass was weighed by using a balance (Sartorius, Germany) with an accuracy of 0.001 g and diluted to a concentration of 100 mg/mL with sterile PBS. 1 μL Dil (Cell membrane red fluorescent staining kit) (Beyotime, China) were added to the 200 μL myelin stock solution and incubated at 37 °C for 1 h to obtain 200 μL fluorescent Dil-myelin with a concentration of 100 mg/mL.

### Preparation of macrophage and myelin conditioned medium

RAW 264.7 cells (Purchased from the Cell Bank of the Chinese Academy of Sciences) were cultured in a six-well plate at a density of 2 × 10^5^ cells/mL with normal DMEM containing 10% FBS for 24 h. Then, the cell culture medium was replaced with serum-free DMEM for another 24 h, and the supernatant was collected as the RAW macrophage conditioned medium after centrifugation at 2000 rpm for 15 min. Myelin conditioned medium was obtained by diluting the myelin stock solution (100 mg/mL) with DMEM to a concentration of 0.5 mg/mL. The medium was preserved at 4 °C and protected from light for a maximum period of 1 week.

### Cell proliferation experiments

Cells were seeded in a 96-well plate at a density of 2000 cells/well. The hBcl2, cb, NC and BMSCs groups were established, with 4 parallel wells set for each group at each time point. After 1, 2, 3, 4, 5, and 6 days of cell culture, 100 μL fresh DMEM was replaced, and 2 μL of Cell Counting Kit-8 (CCK8) (Biosharp, China) solution was added to each well. The plate was then incubated at 37 °C for 4 h. At least three independent replicates were performed for the above experiment. The absorbance at 450 nm was measured using a microplate reader (Molecular Devices, USA).

### Serum deprivation experiment

Cells were seeded in a 96-well plate at a density of 4000 cells/well. The hBcl2, NC, cb, and BMSCs groups were established, with 4 parallel wells set for each group at each time point. After culturing the cells with DMEM containing 10% FBS for 1 day, the DMEM medium without FBS was replaced to continue culturing the cells in each group. After 1, 2, 3, 4, 5, 6, 7 and 8 days of cell culture, CCK8 analysis was performed as mentioned before. At least three independent replicates were performed for the above experiment.

### Western blot analysis

Western blot was performed to confirm the transfection efficiency of lentiviral vectors. Protein samples (20 µg) isolated from each cell group were loaded and separated via 10% sodium SDS-polyacrylamide gel electrophoresis, followed by transfer to polyvinylidene difluoride membranes (Millipore, Bedford, MA, USA). The membranes were blocked with 5% nonfat milk in Tris-buffered saline with 0.5% Tween-20 (TBST) at room temperature for 30 min. After washing the membranes with TBST, they were incubated overnight at 4 °C with mouse anti-β-Tubulin (1:10,000, T0023, Affinity, USA) and mouse anti-hBcl2 antibody (1:100, NBP2-15200, Novus, USA). Then, the membranes were incubated with goat anti-mouse secondary antibody (1:10,000, A4416, Sigma, USA) conjugated with horseradish peroxidase at room temperature for 1 h. Labeled proteins were visualized on the membranes using an enhanced chemiluminescence detection kit (Thermo Fisher Scientific, USA). Finally, Image J (NIH, USA) was used for quantitative analysis. The intensity of the β-Tubulin bands was used for normalization.

### Immunocytochemistry

The cells were fixed in 4% paraformaldehyde for 5 min and blocked with 5% donkey serum albumin at room temperature for 30 min. They were then incubated with the primary antibodies at 4 °C overnight. The primary antibodies used for staining were as follows: mouse anti-hBcl2 (1:100, NBP2-15200, Novus, USA), rabbit anti-cleaved caspase3 (1:100, ab32042, Abcam, USA), and mouse anti-Lamp1 (1:100, sc20011, Santa Cruz, USA). The samples were incubated with secondary antibodies at room temperature for 1 h. The secondary antibodies used were as follows: donkey anti-mouse Alexa Fluor 488, donkey anti-mouse Alexa Fluor 647, donkey anti-mouse Alexa Fluor 594, and donkey anti-rabbit Alexa Fluor 594 (1:500, Termo Fisher Scientific, USA). 4′,6-Diamidino-2-phenylindole dihydrochloride (DAPI, C1005, Beyotime Biotechnology, China) was used for nuclear staining.

### Establishment of the SCI animal model and BMSCs transplantation

Female SD rats were used in the animal experiments. The rats were randomly divided into three groups: (1) NC group (SCI + negative control BMSCs transplantation; n = 3); (2) hBcl2 group (SCI + hBcl2 overexpressing BMSCs transplantation; n = 3); (3) cb group (SCI + BMSCs overexpressing hBcl2-cb transplantation; n = 3). After anesthesia with 2% pentobarbital sodium, rats were subjected to laminectomy at the T9-10 vertebral level. Dumont forceps with a 0.4-mm-wide tip (11223-20, Fine Science Tools, Germany) were used to completely compress the spinal cord from both sides for 10 s, resulting in moderate compression injury^18^. Bladders were emptied manually twice daily, and the rats were allowed free feeding. Three days after SCI, the rats were anesthetized again, and their spinal cords were re-exposed. The cell suspension concentration were adjusted to 2 × 10^4^/μL using a cell counter, and the cell suspension was slowly injected on both sides of the lesion epicenter using a siliconized Hamilton syringe (7634-01 and 7803-05, Hamilton, Switzerland)^19,20^. A volume of 5 μL of cell suspension was injected on each side at a rate of 0.5 μL/min, and the needle was held in place for 5 min before being slowly removed. The muscles and skin were sutured sequentially, and the rats were transferred to the animal house for culture after iodine sterilization.

### Tissue preparation and immunofluorescence staining

Rats were anesthetized with 2% pentobarbital sodium, and the blood was removed by transcardial perfusion with PBS. A 5-mm segment of spinal cord tissue surrounding the injury site was harvested for histological analysis after perfusion with 4% paraformaldehyde. The spinal cord segment was then embedded in OCT embedding agent, and 16 μm sagittal sections were obtained using a microtome (RM2235, Leica, Germany). The sections were blocked in 10% donkey serum containing 0.3% Triton X-100 (SL050 and T8200, Solarbio, China) at room temperature for 1 h, followed by overnight incubation with primary antibodies at 4 °C. The primary antibodies included mouse anti-NF200 (1:500, ab213128, Abcam, USA), rabbit anti-GFAP (1:200, 16825-1-AP, Proteintech, China), mouse anti-hBcl2 (1:100, NBP2-15200, Novus, USA), and rabbit anti-cleaved caspase3 (1:100, ab32042, Abcam, USA). Then, the sections were incubated with secondary antibodies at room-temperature for 1 h. The secondary antibodies used were donkey anti-mouse Alexa Fluor 555, donkey anti-rabbit Alexa Fluor 488, and donkey anti-mouse Alexa Fluor 488, donkey anti- rabbit Alexa Fluor 555 (1:500, Termo Fisher Scientific, USA). Finally, the sections were stained with DAPI to label the nuclei. The images of the sections were acquired using a Zeiss LSM 900 confocal microscope system and a Zeiss Axio Scope A1 fluorescence microscope.

### Statistical analysis

Data were presented as mean ± SD. All experiments were carried out independently at least 3 times. Quantification was conducted using a blind method. Statistical analysis was performed using Graph Pad Prism 5.0 (La Jolla, USA). The unpaired t test was used to analyze the data between two groups. Multiple comparisons were analyzed by one-way or two-way analysis of variance (ANOVA), followed by Tukey’s post hoc test. Values of *P* < 0.05 were considered to have significant differences.

### Ethical approval

All animal experiments were carried out in accordance with institutional animal care protocols and were authorized by Anhui Medical University Ethical Committee (No. LLSC20160052). Adult female Sprague-Dawley (SD) rats were supplied from Experimental Animal Center of Anhui Medical University (No. SCXK (Wan) 2017-001).

### Statement

The study is reported in accordance with ARRIVE guidelines.

## Results

### Identification of rat BMSCs

**Figure 1:**
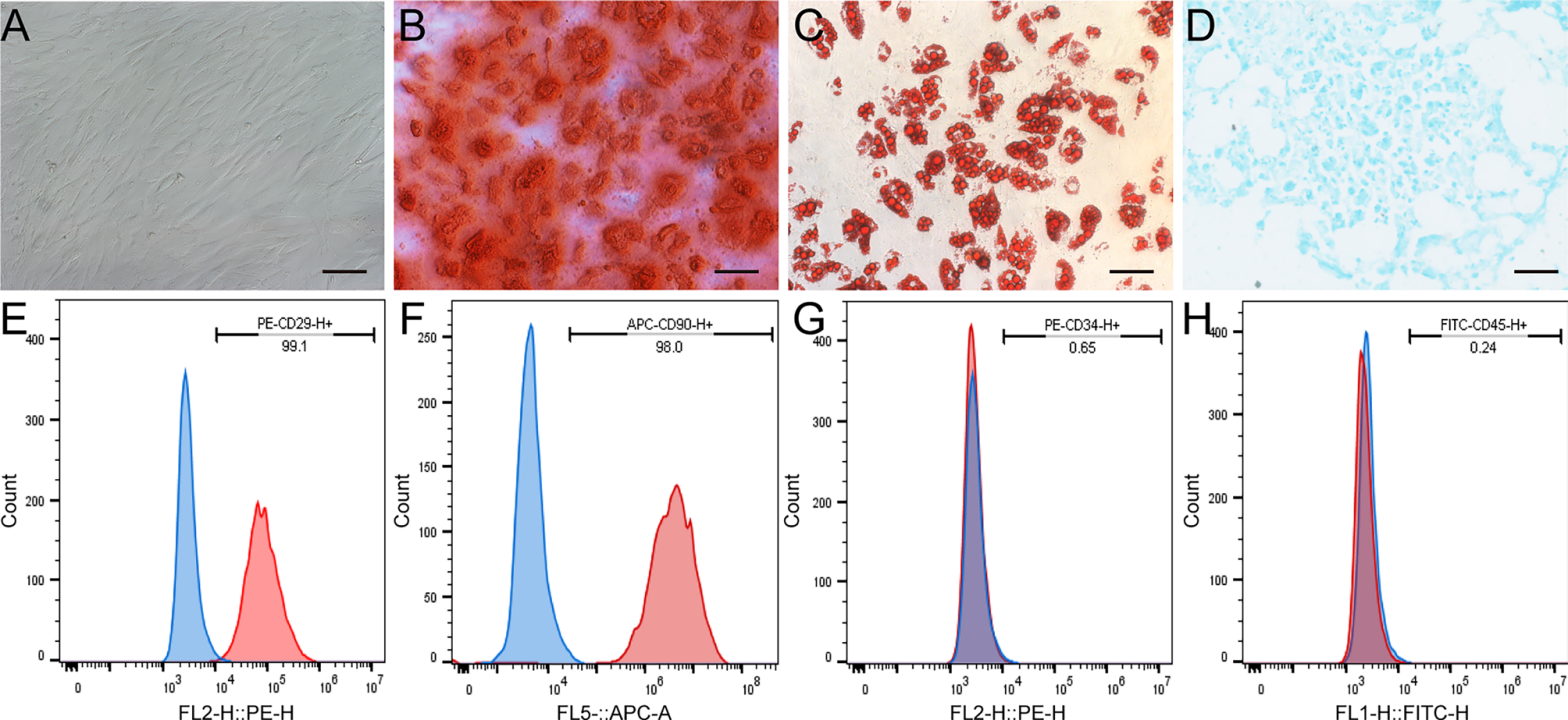
Cell culture and identification of rat BMSCs. (**A**) Phase-contrast imaging showed the primary rat BMSCs cultured in vitro to the third generation. (**B**) Alizarin red staining revealed that BMSCs had osteogenic differentiation ability. (**C**) Oil red staining revealed that BMSCs had adipogenic differentiation ability. (**D**) Allicin blue staining revealed that BMSCs had chondrogenic differentiation ability. (**E**–**H**) Flow cytometry was used to identify the expression of positive markers (CD29^+^ in e, 99.1%, CD90^+^ in f, 98%) and negative markers (CD34^+^ in g, 0.65%, CD45^+^ in h, 0.24%) on the surface of BMSCs. Scale bars = 50 μm (**A**–**D**).

The morphology of rat BMSCs was observed using an inverted microscope, revealing typical spindle-shaped cells (Fig. 1A). Upon reaching a cell fusion degree of 85–95%, the cell gaps disappeared, and the long axes of most cells became parallel to each other. To confirm the stem cell-like properties of the isolated cells, a three-line differentiation kit was used to demonstrate their osteogenic, adipogenic, and chondrogenic differentiation capabilities (Fig. 1B–D). Flow cytometry analysis showed the BMSCs were positive for CD90, CD29, but negative for CD34 and CD45 (Fig. 1E–H). These results provide evidence of the multipotent differentiation potential of the BMSCs and support their suitability for further investigation.

### hBcl2 overexpression in infected BMSCs in vitro

**Figure 2:**
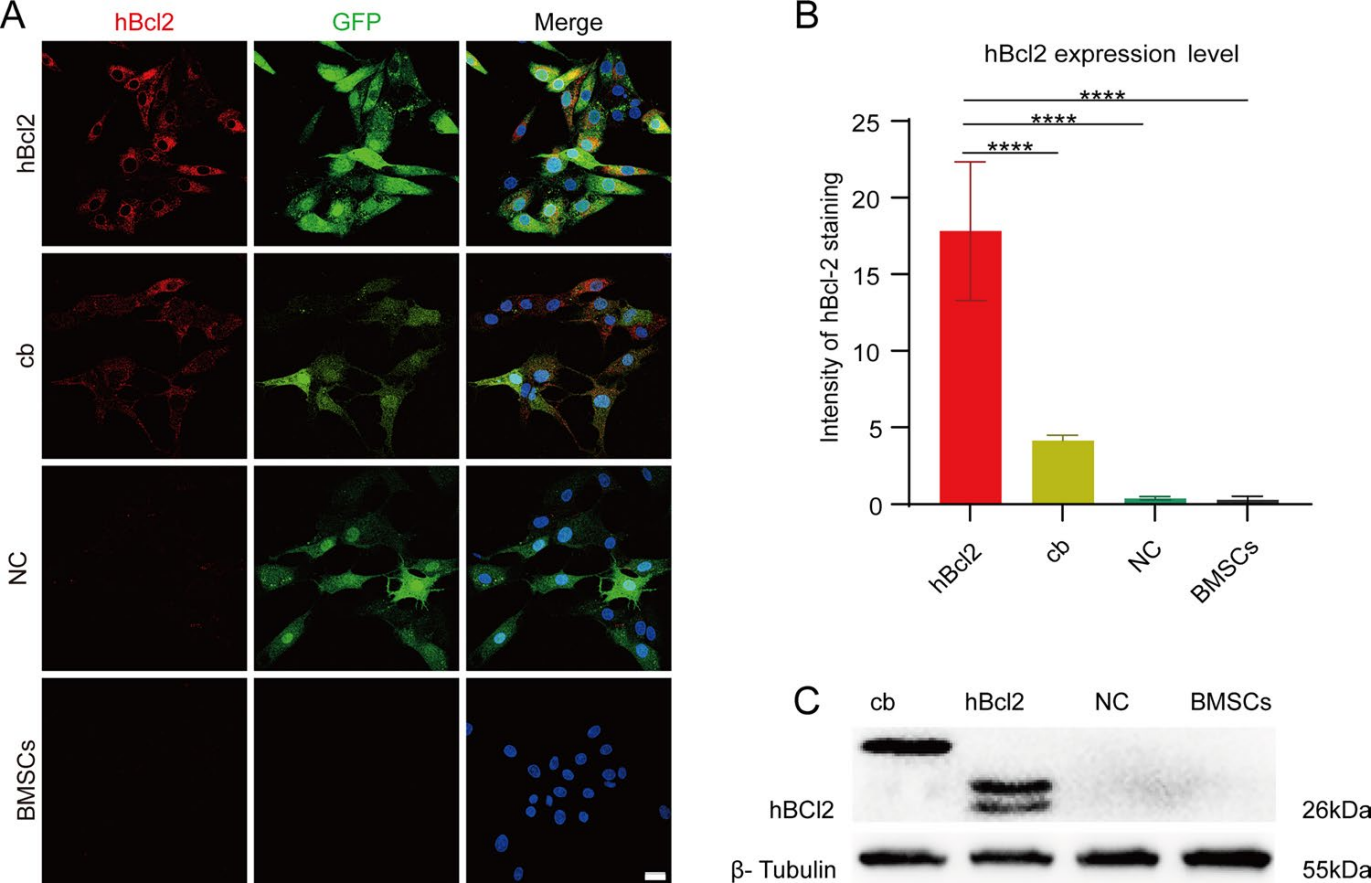
Expression of hBcl2 in different groups of BMSCs models. (**A**) Cellular immunofluorescence staining was used to determine the amounts of hBcl2 protein (red) and GFP protein (green) expression in cells from the hBcl2, cb, NC, and normal BMSCs groups. (**B**) Quantitative analysis of the average fluorescence intensity of hBcl2 expression in each group (At least three independent replicates were performed for each experiment. Data are presented as mean ± SD, ****P < 0.0001 vs. hBcl2 group analyzed by one-way ANOVA, followed by Tukey’s post hoc test). (**C**) Western blot results revealed hBcl2 protein expression in the hBcl2, cb, NC, and BMSCs groups (original blots were presented in Supplementary Fig. 1). Scale bar = 10 μm (**A**).

As mentioned above, BMSCs were infected with lentivirus vectors to create cell models for the hBcl2, cb and NC groups. Immunocytochemistry staining was used to evaluate the BMSCs models that were constructed. As shown in Fig. 2A, successful lentivirus infection was indicated by the presence of GFP^+^ cells in the hBcl2, cb and NC groups, compared to normal BMSCs. Immunocytochemistry staining of hBcl2 showed that hBcl2 protein was expressed in both the hBcl2 and cb groups, with higher expression levels observed in the hBcl2 group (Fig. 2A, B) (At least three independent replicates were performed for each experiment. ****P < 0.0001 vs. hBcl2 group analyzed by one-way ANOVA, followed by Tukey’s post hoc test). Western blot analysis also confirmed successful expression of hBcl2 protein in both the hBcl2 and cb groups (Fig. 2C). Notably, the molecular weight of the positive bands in the cb group was higher due to the addition of the fragment anchored to the endoplasmic reticulum (hBcl2-cb). These results indicate the successful construction of BMSCs models overexpressing hBcl2 and hBcl2-cb.

### Effect of hBcl2 overexpression on cell proliferation and apoptosis

**Figure 3:**
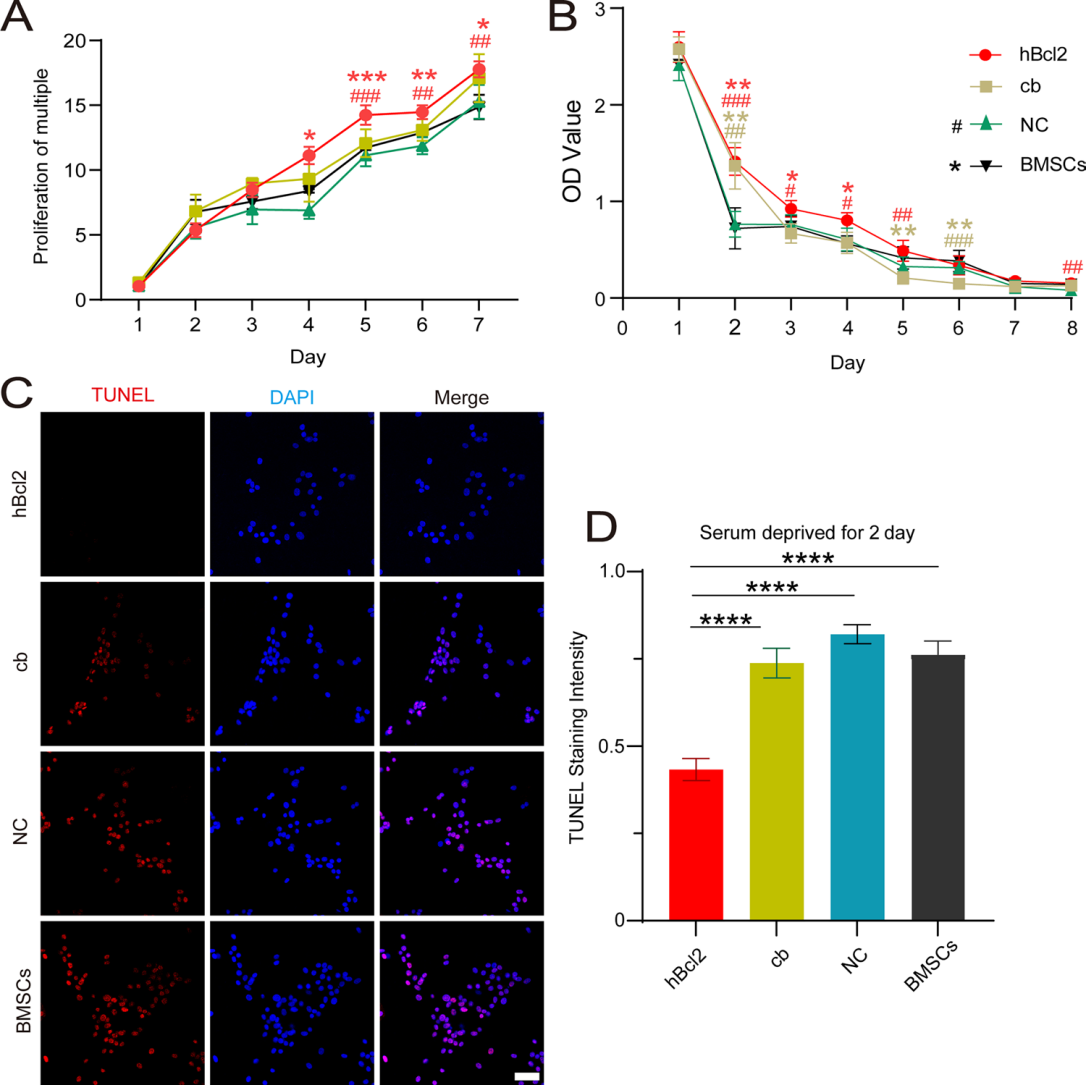
Overexpression of hBcl2 protein can enhance the anti-apoptotic ability of BMSCs in vitro. (**A**) The CCK8 method was used to detect cell proliferation in hBcl2, cb, NC, and BMSCs groups at 1, 2, 3, 4, 5, 6, and 7 days (At least three independent replicates were performed for each experiment. hBcl2 comparisons are shown in red, cb comparisons are shown in gold, BMSC comparisons are shown in black, and NC comparisons are shown in blue. Data are presented as mean ± SD, ***P < 0.001, **P < 0.01, *P < 0.05 vs. BMSC group; ^###^*P* < 0.001, ^##^*P* < 0.01 vs. NC group, analyzed by two-way ANOVA, followed by Tukey’s post hoc test). (**B**) The CCK8 method was used to detect cell survival in each group at 1, 2, 3, 4, 5, 6, 7 and 8 days by using serum deprivation treatment (At least three independent replicates were performed for each experiment. hBcl2 comparisons are shown in red, cb comparisons are shown in gold, BMSC comparisons are shown in black, and NC comparisons are shown in blue. Data are presented as mean ± SD, **P < 0.01, *P < 0.05, vs. BMSC group; ^###^*P* < 0.001, ^##^*P* < 0.01, ^#^*P* < 0.05 vs. NC group, analyzed by two-way ANOVA, followed by Tukey’s post hoc test). (**C**) The apoptosis of cells in each group was measured by TUNEL staining. DAPI (blue) stained nuclei indicating the total number of cells, and TUNEL (red) indicates the apoptotic cells. (**D**) The proportion of TUNEL positive cells in each group (At least three independent replicates were performed for each experiment. ****P < 0.0001 vs. hBcl2 group, analyzed by one-way ANOVA, followed by Tukey’s post hoc test). Scale bar = 50 μm.

To evaluate the impact of hBcl2 overexpression on BMSCs proliferation, we used the CCK8 kit to assess the growth status of BMSCs in different groups under normal culture conditions for 1–7 days. The results revealed that the cell proliferation efficiency in the hBcl2 group was significantly higher than that in the NC group from days 5–7 (Fig. 3A) (At least three independent replicates were performed for each experiment. hBcl2 comparisons are shown in red, cb comparisons are shown in gold, BMSC comparisons are shown in black, and NC comparisons are shown in blue. ^###^*P* < 0.001, ^##^*P* < 0.01, analyzed by two-way ANOVA, followed by Tukey’s post hoc test). Moreover, after 2–6 days of serum-free culture, the survival rate in the hBcl2 group was significantly higher than that in the NC group (Fig. 3B) (^###^*P* < 0.001, ^##^*P* < 0.01, ^#^*P* < 0.05, analyzed by two-way ANOVA, followed by Tukey’s post hoc test). To further investigate the effect of hBcl2 overexpression on apoptosis, we performed TUNEL staining to detect apoptotic cells in each group after 1 day of serum deprivation treatment. Our results showed that the number of TUNEL^+^ apoptotic cells in the hBcl2 group was significantly reduced compared to the cb group, NC group and BMSCs group, while the number of TUNEL^+^ apoptotic cells in the other three groups had no significant statistical difference (Fig. 3C,D) (At least three independent replicates were performed for each experiment. ****P < 0.0001 vs. hBcl2 group, analyzed by one-way ANOVA, followed by Tukey’s post hoc test). These findings indicate that BMSCs overexpressing hBcl2 exhibit significantly enhanced anti-apoptotic ability, while the overexpression of hBcl2 located in the endoplasmic reticulum does not promote the anti-apoptotic response of cells induced by a starvation environment.

### BMSCs overexpressing hBcl2 with enhanced anti-apoptotic ability could resist myelin debris-induced early apoptosis in vitro

**Figure 4:**
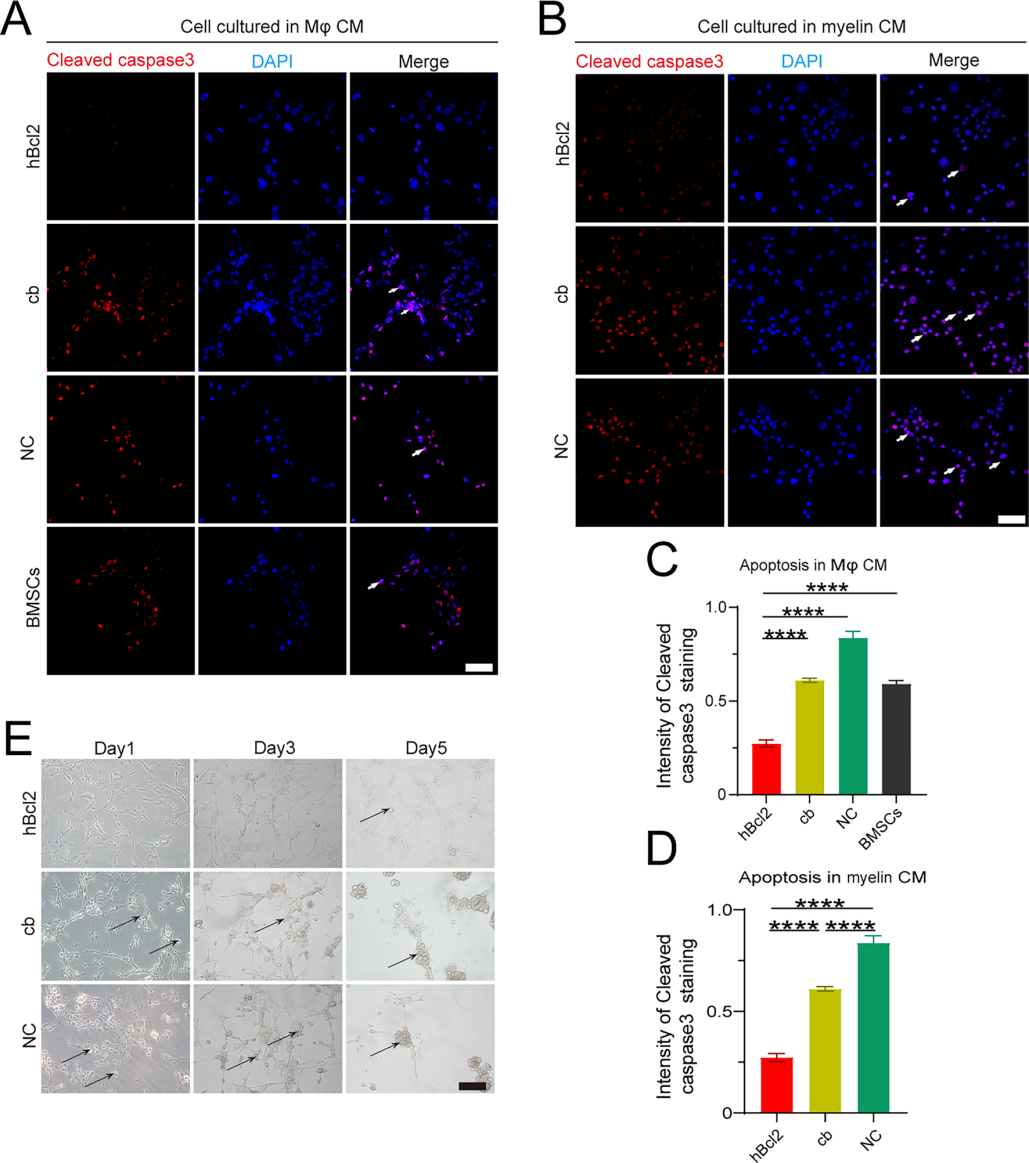
hBcl2 overexpressing BMSCs with enhanced anti-apoptotic ability could resist myelin debris-induced early apoptosis in vitro. (**A**) Cellular immunofluorescence staining of cleaved caspase3 was used to assess apoptosis in the hBcl2, cb, NC, and BMSCs groups after incubating cells with macrophage conditioned medium (Mφ CM) for 24 h. White arrows represent apoptotic cells. (**B**) Cleaved caspase3 immunofluorescence staining was used to determine the apoptosis of cells in each group after 24 h cell culture in Dil-myelin CM (500 μg/mL). (**C**,**D**) Quantitative analysis of the proportion of cleaved caspase3 positive cells in the total number of cells, which were culture in macrophage CM (**C**) or myelin debris (**D**) (At least three independent replicates were performed for each experiment. Data are presented as mean ± SD, ****P < 0.0001 vs. hBcl2 group, cb vs. NC group, analyzed by one-way ANOVA, followed by Tukey’s post hoc test). (**E**) The morphological changes of apoptotic cells (black arrow) were observed under a light microscope after 24 h cell culture in myelin debris (500 μg/mL). Scale bars = 50 μm (**A**,**B**,**E**).

To explore the survival ability of BMSCs overexpressing hBcl2 within an impaired microenvironment, we simulated the inflammatory environment by utilizing macrophage conditioned medium (Mφ CM) and myelin conditioned medium (Myelin CM). We measured apoptotic cells in each group by cleaved caspase3 immunofluorescence staining and observed a significant decrease in the percentage of cleaved caspase3^+^ apoptotic cells in the hBcl2 group (Fig. 4A–D) (At least three independent replicates were performed for each experiment. ****P < 0.0001 vs. hBcl2 group, cb vs. NC group, analyzed by one-way ANOVA, followed by Tukey’s post hoc test). Furthermore, we observed the morphology of the cells in each group under an optical microscope after 1, 3 and 5 days of culture in Myelin CM. We found that cells in the cb and NC groups exhibited bright death morphology on day 1 and clumping on days 3 and 5. In contrast, the hBcl2 group maintained a normal cell morphology until day 5 (Fig. 4E). These results demonstrate that BMSCs overexpressing hBcl2 with enhanced anti-apoptotic ability could resist myelin debris-induced early apoptosis in vitro*.*

### BMSCs can phagocytize and process myelin debris under the mediation of lysosomes

**Figure 5:**
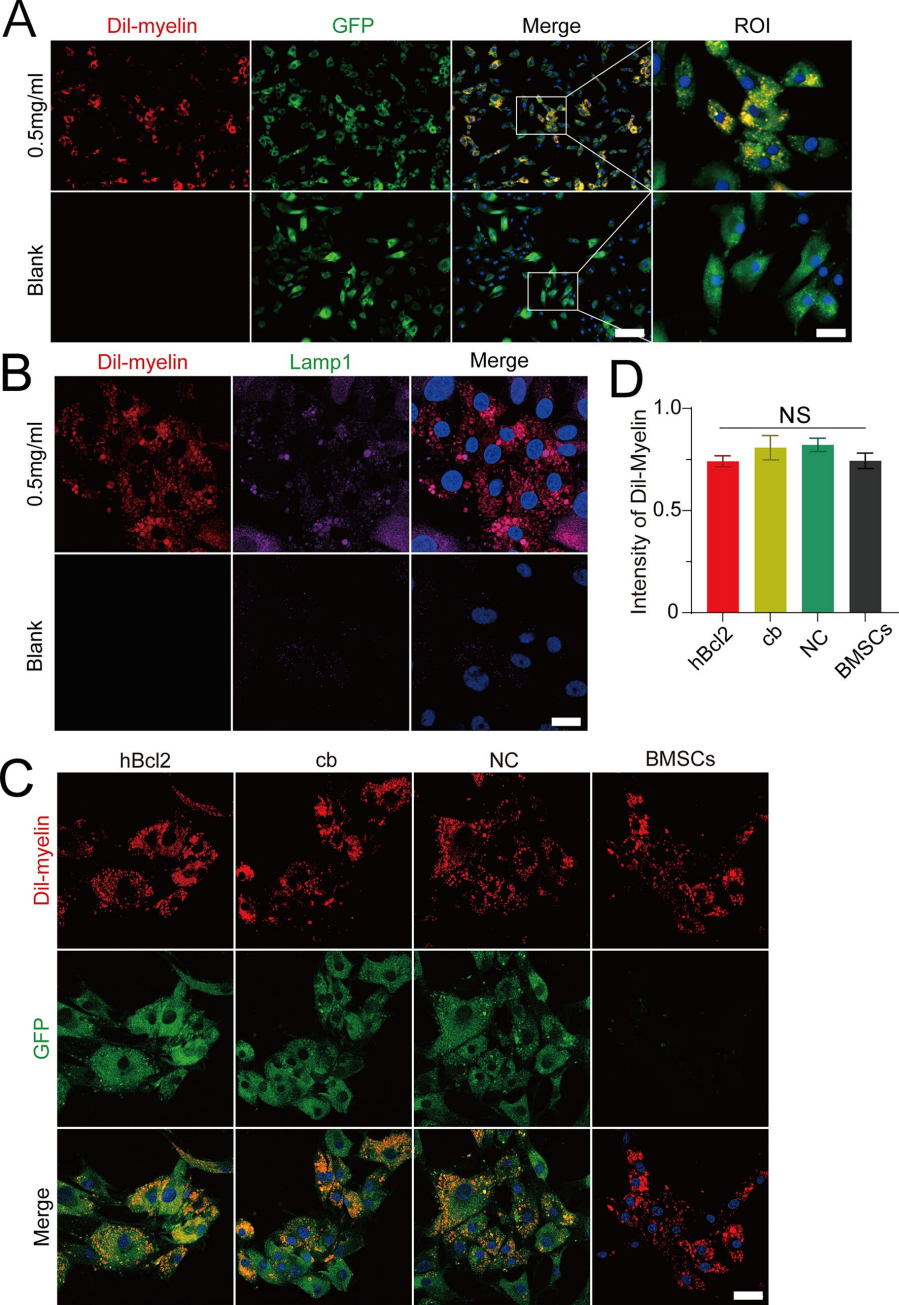
BMSCs can phagocytose and process myelin debris via the lysosomal pathway. (**A**) Immunofluorescence labeling was used to assess the phagocytic capacity of BMSCs (GFP, green) in the hBcl2 group to Dil-myelin (red, 500 μg/mL). Under a high-powered microscope, GFP and Dil labeling revealed that BMSCs in the hBcl2 group could phagocytize myelin. (**B**) Cellular immunofluorescence staining was used to assess changes of Lamp1 (green) in BMSCs following phagocytosis of Dil-myelin (red, 500 μg/mL). The number of cells is shown by DAPI. After phagocytosis of myelin, Lamp1 expression rose considerably in all BMSCs. (**C**) After adding Dil-myelin (red, 500 μg/mL) for 1 day, a double fluorescence staining was used to determine the phagocytosis of Dil-myelin (red, 500 μg/mL) by cells in the hBcl2, cb, NC, and BMSCs groups. (**D**) Quantitative analysis of the proportion of cells with Dil-myelin (red, 500 μg/mL) phagocytized by BMSCs in each group accounting for 1/3 or more of the total. (At least three independent replicates were performed for each experiment. Data are presented as mean ± SD, *NS*, no statistical difference, analyzed by one-way ANOVA), scale bar = 100 μm (**A**), scale bars = 20 μm (ROI, **C**), scale bar = 10 μm (**B**).

To determine whether BMSCs are capable of phagocytizing myelin debris, we added Dil-myelin to the cells in the hBcl2 group and set up a control group with blank medium. Immunocytochemistry results revealed the presence of red granular myelin debris within the Dil-myelin group cells after 1 day of co-culturing (Fig. 5A). We then detected the expression of Lamp1 in the two groups. Immunocytochemistry results showed that the hBcl2 group exhibited higher Lampl expression compared to the blank control group (Fig. 5B). This indicates the involvement of lysosomes in the process of cell phagocytosis of myelin debris.

To further test the effect of hBcl2 overexpression on myelin phagocytosis of BMSCs, Dil-myelin was added to the hBcl2, cb, NC and BMSCs groups for 1 day. We found that cells in each group were capable of phagocytizing myelin debris, as detected by immunocytochemistry (Fig. 5C). However, statistical analysis showed no significant difference in the proportion of myelin debris-phagocytic cells among the different groups, indicating that overexpression of hBcl2 had no effect on the myelin debris-phagocytic ability of BMSCs (Fig. 5D) (At least three independent replicates were performed for each experiment. NS, no statistical difference, analyzed by one-way ANOVA). These results demonstrate that BMSCs can effectively phagocytize and process myelin debris under the mediation of lysosomes.

### BMSCs overexpressing hBcl-2 promote cell survival by degrading myelin debris through the lysosomal pathway

**Figure 6:**
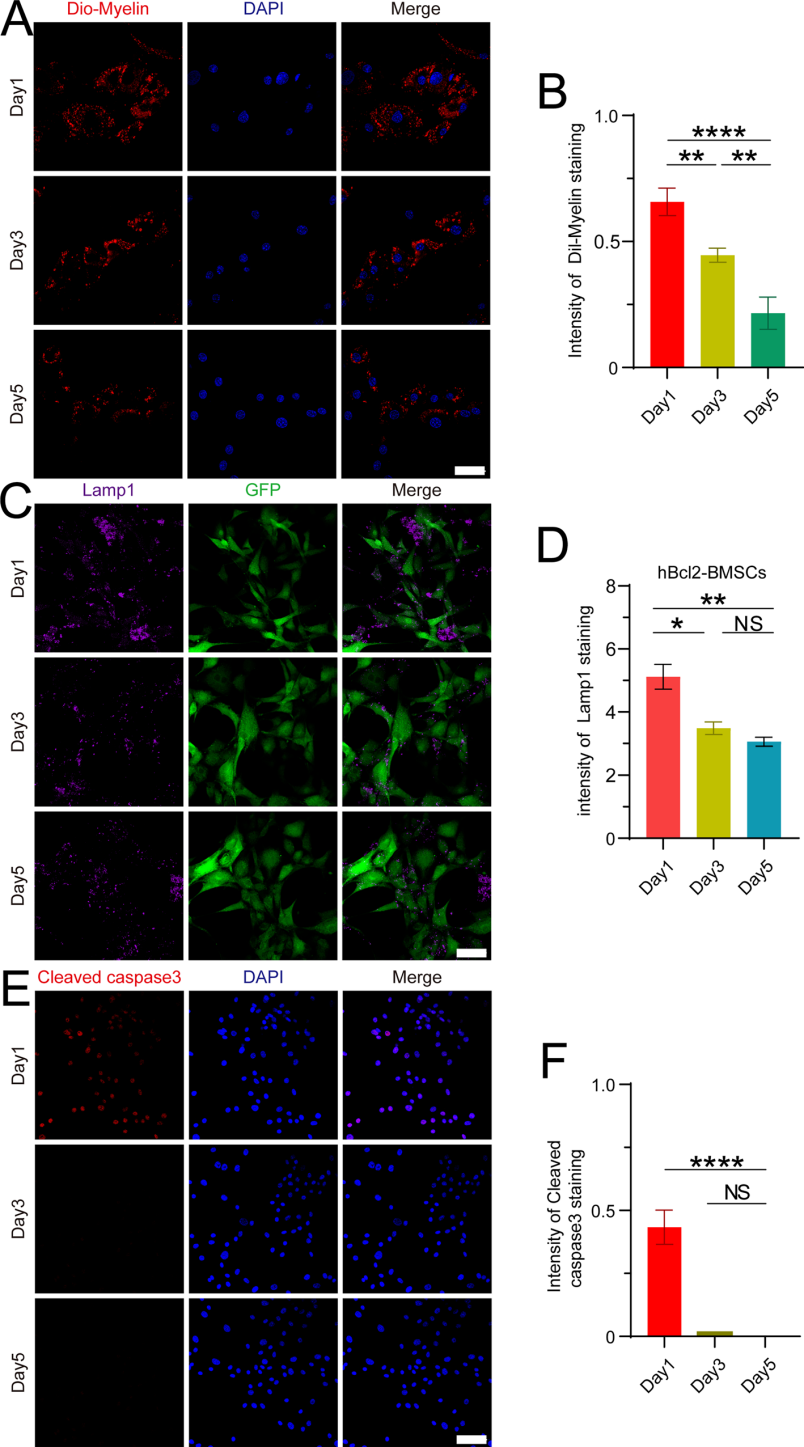
BMSCs overexpressing hBcl2 could effectively degrade myelin debris through the lysosomal pathway to promote cell survival. (**A**) Immunofluorescence staining was used to measure Dil-myelin (red, 500 μg/mL) phagocytosis by BMSCs in the hBcl2 group at 1, 3, and 5 days after treatment with Dil-myelin (red, 500 μg/mL) CM, and all cell nuclei were labeled with DAPI (blue). (**B**) Quantitative analysis of the proportion of cells in which Dil-myelin (red, 500 μg/mL) accounted for 1/3 of the total cell volume and more in the total cell number swallowed by BMSCs in the hBcl2 group at 1, 3, and 5 days after treatment with Dil-myelin (red, 500 μg/mL) CM (At least three independent replicates were performed for each experiment. Data are presented as mean ± SD, ****P < 0.0001, **P < 0.01, Day1 vs. Day3, Day1 vs. Day5 analyzed by one-way ANOVA, followed by Tukey’s post hoc test). (**C**) At 1, 3, and 5 days after treatment with Dil-myelin (red, 500 μg/mL) CM, immunofluorescence labeling was employed to determine the activation of BMSCs (GFP, green) and lysosomes (Lamp1, purple) in the hBcl2 group. (**D**) Quantitative analysis of BMSCs in the hBcl2 group's lysosomal (Lamp1, purple) fluorescence intensity after treatment with Dil-myelin CM at 1, 3, and 5 days. (At least three independent replicates were performed for each experiment. Data are presented as mean ± SD, *P < 0.05, **P < 0.01, vs. Day1, *NS*, no significant difference, Day3 vs. Day5 analyzed by one-way ANOVA, followed by Tukey’s post hoc test). (**E**) Apoptosis was measured by cleaved caspase3 immunofluorescence labeling of BMSCs in the hBcl2 group at 1, 3 and 5 days following treatment with Dil-myelin CM. (**F**) quantification of the proportion of cleaved caspase3 positive cells in the total number of cells (At least three independent replicates were performed for each experiment. Data are presented as mean ± SD, ****P < 0.0001, vs. Day1, *NS*, no significant difference, Day3 vs. Day5 analyzed by one-way ANOVA, followed by Tukey’s post hoc test). Scale bars = 20 μm (**A**,**C**), scale bar = 50 μm (**E**).

To explore the mechanism by which the hBcl2 group cells enhance their anti-apoptosis ability, we treated these cells with Dil-myelin conditioned medium for 1, 3 and 5 days and assessed the phagocytosis of Dil-myelin through immunofluorescence staining. The results demonstrated a gradual decrease in the ratio of cells phagocytozing Dil-myelin to the total number of cells over time (Fig. 6A,B) (At least three independent replicates were performed for each experiment. ****P < 0.0001, **P < 0.01, Day1 vs. Day3, Day1 vs. Day5 analyzed by one-way ANOVA, followed by Tukey’s post hoc test), which was consistent with changes observed in Lamp1 expression (Fig. 6C,D) (At least three independent replicates were performed for each experiment. *P < 0.05, **P < 0.01, vs. Day1, *NS*, no significant difference, Day3 vs. Day5 analyzed by one-way ANOVA, followed by Tukey’s post hoc test). Immunocytochemistry staining of cleaved caspase3 revealed a significantly lower number of apoptotic cells at 3 and 5 days compared to 1 day (Fig. 6E,F) (At least three independent replicates were performed for each experiment. *P < 0.05, ****P < 0.0001, vs. Day1, *NS*, no significant difference, Day3 vs. Day5 analyzed by one-way ANOVA, followed by Tukey’s post hoc test). These results provide evidence that BMSCs overexpressing hBcl2 may promote cell survival by degrading myelin debris through the lysosomal pathway.

### Overexpression of hBcl2 enhances the survival of BMSCs in the injured microenvironment and promotes SCI repair

**Figure 7:**
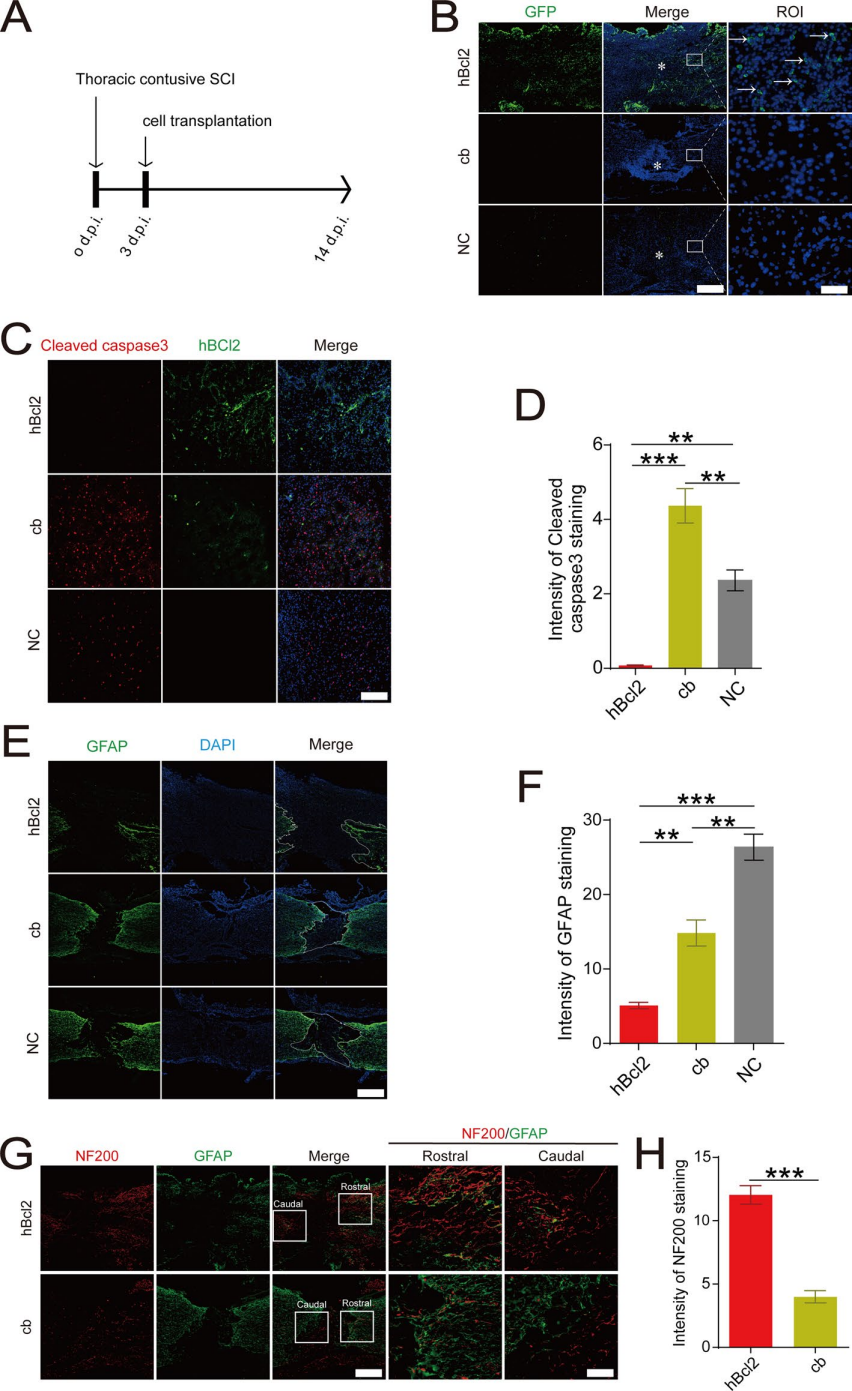
Overexpression of hBcl2 enhances the survival of BMSCs in the adverse microenvironment and promotes the repair of the injured spinal cord. (**A**) Schematic representation. BMSCs in the hBcl2, cb, and NC groups were transplanted in situ 3 days after SCI, and samples were collected 11 days later. (**B**) A spinal cord sagittal fluorescence labelling was used to determine the survival and distribution of BMSCs (GFP, green) in the hBcl2, cb, and NC transplantation groups 14 days post injury (d.p.i.). The arrows show the survived transplanting BMSCs, the asterisks denote the damage sites, and DAPI denotes all viable cells, scale bar = 200 μm (left), scale bar = 20 μm (right); (**C**) The apoptosis of BMSCs transplanted rats at the injury sites at 14 d.p.i. was evaluated by using spinal cord sagittal immunofluorescence staining. Dead cells were designated by cleaved caspase3 positive cells (red), BMSCs overexpressing hBcl2 protein in the transplanted area were labeled by hBcl2 protein (green), and nuclei were labeled by DAPI, scale bar = 100 μm. (**D**) Quantification the intensity of cleaved caspase3 staining in the spinal cord segment spanning the injured core at 14 d.p.i. (At least three independent replicates were performed for each experiment. Data are presented as mean ± SD, **P < 0.01, ***P < 0.001, vs. hBcl2, analyzed by one-way ANOVA, followed by Tukey’s post hoc test). (**E**) Spinal cord sagittal immunofluorescence was used to evaluate the expression and distribution of GFAP in BMSCs transplanted rats in each transplantation group, scale bar = 200 μm. (**F**) Quantification the intensity of GFAP staining in the spinal cord segment spanning the injured core at 14 d.p.i. (At least three independent replicates were performed for each experiment. Data are presented as mean ± SD, ***P < 0.001, **P < 0.01, vs. hBcl2, analyzed by one-way ANOVA, followed by Tukey’s post hoc test). (**G**) In the injured core of rats in the hBcl2 and cb BMSCs transplantation groups, the formation of glial scar (GFAP, green) and the regeneration and preservation of neuronal axons (NF200, red) were measured using immunofluorescence staining. Scale bar = 200 μm (left), scale bar = 100 μm (right). (**H**) Quantification the intensity of NF200 staining in the spinal cord segment spanning the injured core at 14 d.p.i. (Three independent replicates were performed in the experiment. Data are presented as mean ± SD, **P < 0.01, analyzed by *t*-test).

To investigate the impact of BMSCs overexpressing hBcl2 on functional recovery after SCI, we labeled the transplanted BMSCs with lentivirus overexpressing GFP protein at the injury sites (Fig. 7A). The hBcl2 transplantation group exhibited positive expression of GFP in the transplantation area at 14 days post injury, indicating the survival of the transplanted BMSCs in the rat SCI microenvironment. In contrast, no GFP^+^ cells were detected in the NC and cb transplantation groups, suggesting poor survival of the transplanted cells (Fig. 7B). Moreover, the number of cleaved caspase3^+^ cells in the transplanted area of the hBcl2 group was significantly reduced compared to the NC and cb groups (Fig. 7C,D) (At least three independent replicates were performed for each experiment. **P < 0.01, ***P < 0.001, vs. hBcl2, analyzed by one-way ANOVA, followed by Tukey’s post hoc test). These finding suggest that overexpression of hBcl2 enhances the survival of BMSCs at the injury site, improving their pro-recovery effect.

Furthermore, we evaluated the formation of glial scarring in each group. GFAP staining revealed that in the NC and cb transplantation groups, a compact and dense astrocytic scar border formed around the lesion center. However, in the hBcl2 transplantation group, the GFAP^+^ region was loosely structured, indicating reduced glial scarring (Fig. 7E,F). (At least three independent replicates were performed for each experiment. ***P < 0.001, **P < 0.01, vs. hBcl2, analyzed by one-way ANOVA, followed by Tukey’s post hoc test). Double staining of NF200 with GFAP showed that the cb transplantation group exhibited a significant reduction in the number of NF200^+^ neuronal fibers, accompanied by the formation of a dense glial scar in the transplantation area. In contrast, the hBcl2 transplantation group showed an increase in the number of NF200^+^ neuronal fibers, which were neatly arranged and would be beneficial to extend to the injured core through the loose glial scar area (Fig. 7G,H) (Three independent replicates were performed in the experiment, ***P < 0.001, analyzed by *t*-test). In conclusion, our results demonstrate that overexpression of hBcl2 enhances the survival of BMSCs in the SCI microenvironment, which is beneficial to inhibit the formation of dense glial scar and promote axonal preservation in the injured rat spinal cord.

## Discussion

In this study, we investigated the role of hBcl2 overexpression in BMSCs transplantation for SCI treatment. Our results demonstrated that overexpression of hBcl2, not hBcl2-cb, can improve the impact of BMSCs against myelin debris-induced apoptosis, increase the survival rate of transplanted BMSCs, and enhance axonal preservation in the injured spinal cord of rats.

BMSCs have been recognized as “small molecular factories” with neuroprotective properties. Transplantation of BMSCs has been shown to promote endogenous neurorestorative mechanisms, leading to reduced apoptotic cell death and facilitating remodeling of glial, neuronal, and vascular elements^21^. Previous studies by Yuan-Shan Zeng et al. demonstrated that co-seeding of BMSCs with genetically enhanced expression of neurotrophin-3 and its high-affinity receptor tropomyosin receptor kinase C within a three-dimensional gelatin sponge scaffold promoted the differentiation of BMSCs into neural-like cells^22^. Transplantation of these engineered tissues into completely severed spinal cords resulted in the differentiation of BMSCs into neurons and myelin-forming cells, ultimately leading to axon regeneration and functional recovery after SCI^22^. However, conflicting findings have been reported regarding the long-term survival and differentiation of transplanted BMSCs. Kevin Kempet al. suggested that the presence of cell fusion based on the detection of increased bi-nucleated neurons after BMSCs transplantation^23^. Additionally, some studies have reported the disappearance of transplanted BMSCs in the acute or subacute phase after injury due to the inflammatory response occurring 1–2 weeks post-transplantation^7,24^. The effectiveness of BMSCs transplantation in SCI treatment relies on the long-term survival and differentiation of BMSCs^25–27^. Our previous studies have shown that overexpression of tropomyosin receptor kinase A (TrkA), a nerve growth factor receptor, significantly promotes the survival and differentiation of BMSCs into Schwann-like cells in nerve transplantation^16^. This, in turn, facilitates peripheral nerve regeneration and functional recovery^28^. However, due to the high molecular weight of TrkA, gene modification of TrkA does not seem to positively affect the proliferation of BMSCs. Notably, the function of TrkA is closely related to the upregulation of Bcl2 expression^15^. Therefore, in this study, we constructed a BMSC model modified with a lower molecular weight hBcl2 gene. Our findings revealed that overexpression of hBcl2 significantly enhanced the proliferative activity and anti-apoptotic ability of BMSCs. Furthermore, when transplanted into rats with SCI, BMSCs overexpressing hBcl2 exhibited higher survival rates and improved the preservation of axons. These results suggest that BMSCs overexpressing hBcl2 can effectively inhibit apoptosis and promote axonal preservation in SCI.

Myelin debris is one of the main products of necrotic tissue after SCI. Acute SCI leads to vasculature rupture, tissue ischemia, breakdown of the blood-spinal cord barrier, and the accumulation of myelin and neuron debris. Myelin damage occurs immediately after SCI, with cellular debris present in both the surrounding and core regions of the injury within 24 h^2^. Myelin sheaths are primarily composed of lipids (70–85%) and proteins (15–30%), including myelin basic protein (MBP), myelin-associated glycoprotein (MAG), and myelin oligodendrocyte glycoprotein (MOG)^29^. The amount of myelin debris increases in the acute SCI and persists in the chronically injured spinal cord^2^. Previous studies have demonstrated that the abnormal accumulation of myelin debris can have several detrimental effects. Firstly, it directly inhibits the formation of growth cones at the end of axons, impeding axon regeneration^30^. Secondly, it inhibits the maturation and differentiation of oligodendrocytes, preventing myelin regeneration^31^. Thirdly, it stimulates the release of proinflammatory factors by vascular endothelial cells, exacerbating macrophage infiltration^32^. Lastly, our previous research has shown that myelin debris can disrupt the tight connections between endothelial cells in blood vessels, leading to persistent leakage of the blood-spinal cord barrier^17^. Collectively, these factors contribute to a disastrous outcome, severely impeding nerve regeneration and functional recovery. Currently, there is no report on whether BMSCs can phagocytose and clear myelin debris, and how this process affects the survival of BMSCs. In our present study, we discovered that myelin debris induces early apoptosis of normal BMSCs in vitro. However, BMSCs overexpressing hBcl2 can degrade myelin debris through the lysosomal pathway, promoting cell survival. Understanding the mechanisms by which BMSCs interact with and clear myelin debris will provide valuable insights into their therapeutic potential for SCI.

Bcl2 is a key effector in the Bcl2 family that regulates apoptosis^33^. Bc12 is also involved in the regulation of neural differentiation, as overexpression of Bc12 can promote cell differentiation towards a neural lineage^14,34^. Studies on cerebral ischemia in rats have shown that overexpression of Bc12 not only improves the survival of cortical neurons^35^, but also significantly promotes the differentiation of transplanted embryonic stem cells into nerve cells, leading to enhanced functional recovery^34^. Furthermore, Bc12 plays a key role in axon growth and regeneration. In the mammalian central nervous system, while most neurons lose the ability to regenerate severed axons after a certain stage of development, Bcl2 has been found to promote the regeneration of detached axons^36^. Bc12, which is localized in the endoplasmic reticulum, differs from other family proteins. Research by Dong Feng Chen et al. suggests that Bcl2 with an endoplasmic reticulum-anchored cb segment (Bcl2-cb) supports axonal growth by enhancing intracellular Ca^2+^ signaling, extracellular-regulated kinase (Erk) and cyclic adenosine monophosphate response element binding protein (CREB) activation, which in turn stimulate the regenerative response^14^. On the other hand, Bcl-x(L), which is mainly located in in the cytoplasm and mitochondrial outer membrane, does not activate CREB or Erk, nor does it promote axon regeneration^37^. Therefore, Bc12 can not only promote cell survival and neuronal differentiation but also facilitate axon regeneration through the Ca^2+^-Erk/CREB pathway. We initially anticipated that the hBcl2-cb group could potentially exhibit dual benefits by both preventing apoptosis and promoting neural differentiation. Surprisingly, we found that overexpression of hBcl2-cb, which localizes to the endoplasmic reticulum, did not promote cell survival under starvation conditions and within the transplants. However, we observed that overexpression of hBcl2 significantly enhanced the anti-apoptotic ability and increased the survival rate of transplanted BMSCs, as well as improved axonal preservation in the injured rat spinal cord. This may be attributed to the fact that the modification of hBcl2-cb results in excessive binding to the ER, which could potentially limit its anti-apoptotic function. Mobilization of endoplasmic reticulum calcium stores triggers the activation of cytoplasmic death pathways and sensitizes mitochondria to direct proapoptotic stimuli^38^. It is also possible that hBcl2 inhibits Beclin1-induced cellular autophagy when anchored to the endoplasmic reticulum^39^. In the context of SCI, ensuring the survival of transplanted cells is top priority. Once cell survival is secured, the neural differentiation function of the cells can be further explored. The overall effect of treatment with transplantation of BMSCs overexpressing hBcl2 was therefore the rescue of neuronal cells and axons by modulating myelin debris phagocytosis and reducing apoptosis.

In this study, the precise subcellular location of hBcl2 has not been conclusively determined. It is possible that overexpression of hBcl2-cb is anchored in the ER, while overexpression of hBcl2 is located on both the surface of the ER and mitochondria. Alternatively, subtle structural disparities between these proteins may confer an additional signal for hBcl2-cb to attach to the ER. Moreover, our study demonstrated that transplantation of BMSCs overexpressing hBcl2 promotes cell survival by degrading myelin debris through the lysosomal pathway, thereby improving functional recovery after SCI. However, the specific molecular mechanisms involved in this process have not been fully elucidated. Given the complexity of SCI pathology, it is evident that a singular signaling pathway may be insufficient to comprehensively explicate this complex process. There may be other signaling pathways involved, which require further investigation. Furthermore, while our histopathological analysis revealed that treatment with transplantation of BMSCs overexpressing hBcl2 was beneficial to axonal regeneration in vivo, further exploration is needed to determine if the beneficial effects are dose-dependent. Additionally, more detailed data on functional recovery in animal models should be explored in future studies.

## Conclusions

In summary, our study provides evidence that hBcl2 overexpression plays a pivotal role in enhancing the efficacy of BMSCs against apoptosis induced by myelin debris. It increases the survival of transplanted BMSCs and enhances axonal preservation in the injured spinal cord. Consequently, the overexpression of hBcl2 represents a promising strategy for improving the therapeutic potential of clinical BMSC transplantation procedures.

### Supplementary Information

Supplementary Figure 1.

## Data Availability

All data generated or analysed during this study are included in this published article [and its supplementary information files].
