# Supplementary material for: hBcl2 overexpression in BMSCs enhances resistance to myelin debris-induced apoptosis and facilitates neuroprotection after spinal cord injury in rats
Source: Sci Rep. 2024 Jan 21;14:1830. doi: 10.1038/s41598-024-52167-4 (PMC10800342; doi:10.1038/s41598-024-52167-4)
Supplement: Supplementary file 1 — Supplementary Figure 1. [file 41598_2024_52167_MOESM1_ESM.docx]

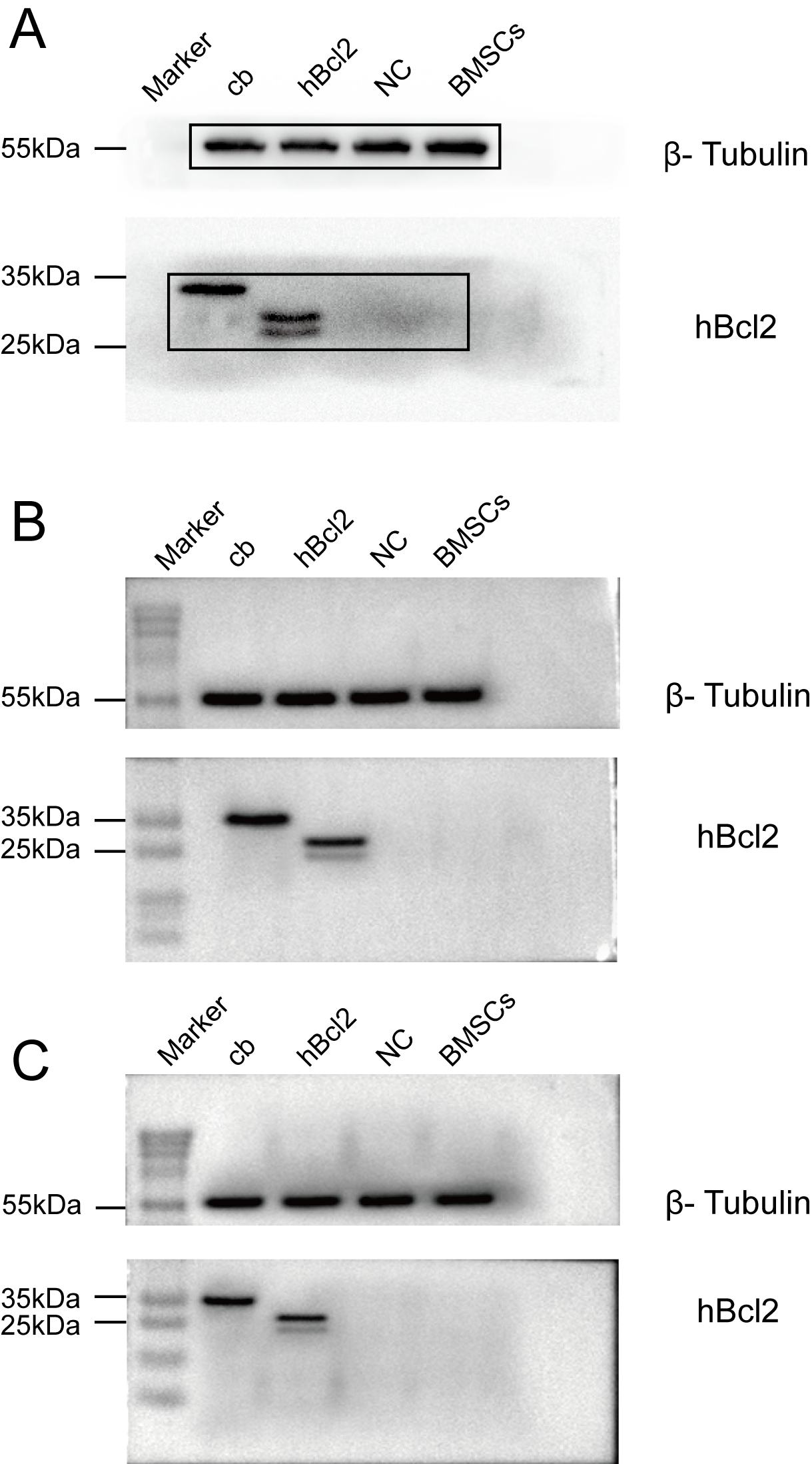


**Supplementary Figure 1** (A) Original immunoblots of hBcl2 and β-Tublulin proteins in the hBcl2, cb, NC, and BMSCs groups. Prior to hybridisation with antibodies during blotting, the blots were cut according to the molecular sizes marked by the PageRuler prestained protein ladder (#26616, Thermo Fisher Scientific, United States). The black boxes indicate the cropped blots as presented in Figure 2C. (B, C) Two independent replicates were performed in the experiment. The original images have been provided to display the full-length membranes with visible edges.
